# Development, Physicochemical Characterization, and Biological Activity of a Gel Based on *Iris scariosa* L. Extract

**DOI:** 10.3390/molecules31101591

**Published:** 2026-05-09

**Authors:** Ailazzat A. Aitkenova, Gayane A. Atazhanova, Karakoz Z. Badekova, Aleksandr V. Samorodov, Saule B. Akhmetova, Gulmira B. Shapatova, Anar K. Brazhanova, Assemay O. Imanbek, Elena A. Smolyarchuk, Bibigul B. Ashirbekova

**Affiliations:** 1 School of Pharmacy, Karaganda Medical University, Karaganda 100017, Kazakhstan; a.aytkenova@qmu.kz (A.A.A.);; 2 Department of Pharmacology, Sechenov First Moscow State Medical University, Moscow 119435, Russia; avsamorodov@gmail.com (A.V.S.);; 3 Department of Biomedicine, Karaganda Medical University, Karaganda 100017, Kazakhstan; 4 Department of Morphology, Karaganda Medical University, Karaganda 100017, Kazakhstan; 5 Department of Informatics and Biostatistics, Karaganda Medical University, Karaganda 100017, Kazakhstan

**Keywords:** *Iris scariosa*, rhizome extract, essential oil, gel formulation, antioxidant activity, anti-inflammatory activity

## Abstract

The aim of this study was to develop and characterize a soft gel dosage form based on an ethanol extract and essential oil from the rhizomes of *Iris scariosa* L., and to evaluate its antioxidant and anti-inflammatory activity. Plant material was extracted using ultrasonic extraction with ethanol of varying concentrations. Phytochemical composition was analyzed by HPLC and GC-MS. Carbomer-based gels were formulated and assessed for physicochemical properties, rheological behavior, stability, and release of active compounds. The highest extraction yield (21.16%) was achieved with 70% ethanol. The extract contained phenolic compounds, mainly epicatechin and gallic acid, as well as flavonoids such as naringin and phlorizin. The essential oil was rich in medium-chain fatty acids. The developed gels were transparent, homogeneous, and exhibited stable pH values within the physiological skin range, along with pseudoplastic flow behavior. Significant antioxidant activity was observed in both the extract and gel in reactive oxygen species and lipid peroxidation models. In a formalin-induced inflammation model, the gel significantly reduced paw edema. These findings demonstrate the potential of the *Iris scariosa*-based gel as a promising phytopharmaceutical formulation for topical application.

## 1. Introduction

In recent years, pharmaceutical research has increasingly focused on medicinal plants as a source of biologically active compounds with multidirectional pharmacological effects. Plant extracts are highly biocompatible, well tolerated, and, as a rule, cause fewer adverse reactions than synthetic substances [1,2]. Their pharmacological value is due to their multidirectional action associated with the presence of phenolic compounds, as well as the possibility of local application with minimal systemic load [3]. The relevance of phytotherapy is confirmed by healthcare practice. According to the World Health Organization, more than 75% of the world’s population uses medicinal plants in primary health care [4]. In this context, the flora of Kazakhstan, characterized by high biodiversity, represents a significant resource for the development of original herbal preparations.

The therapeutic efficacy of plant extracts is determined not only by their chemical composition, but also by their dosage form. Modern pharmaceutical technology is focused on creating delivery systems that ensure stability, controlled release, and localized action of active substances [5,6]. In this regard, soft dosage forms for external use are of particular interest.

Soft dosage forms are characterized by a viscoplastic consistency, structural homogeneity, and the ability to provide a pronounced local effect with minimal systemic load [7]. These properties make them promising carriers of plant extracts for inflammatory and dermatological diseases. Among soft dosage forms, gels are considered the most technologically versatile platform. According to pharmacopoeial definitions, gels are homogeneous semi-solid systems with a three-dimensional polymer matrix [8,9,10]. Their physicochemical and rheological characteristics are regulated by the composition of the base and the nature of the gelling agent. This structural organization ensures uniform distribution of active substances, increased bioavailability, and the possibility of controlled release [11,12]. These advantages are particularly significant when using plant extracts rich in secondary metabolites with anti-inflammatory, antimicrobial, and antioxidant activity [11].

The development of gel dosage forms requires compliance with pharmacopoeial and regulatory requirements, including control of homogeneity, microbiological purity, preservative efficacy, and compatibility with packaging [8]. The quality of the drug is determined at all stages of its life cycle in accordance with the principles of good manufacturing practice (GMP) [4]. Modern approaches to pharmaceutical development, based on ICH Q8–Q10 recommendations, ensure the integration of quality, safety, and efficacy requirements into a unified risk management system [13,14,15]. This is especially important when creating soft dosage forms of plant origin.

Plants of the genus *Iris* L. have long been used to treat inflammatory diseases, skin lesions, and infections, with the rhizomes of *I. germanica* traditionally used for dermatological inflammations, ulcers, and to ease teething in children [16]. Modern studies have confirmed that members of the *Iris* genus contain flavonoids, isoflavonoids, and xanthone compounds capable of suppressing inflammatory mediators, including the production of nitric oxide (NO) induced by LPS, and that methanol extracts of certain species exhibit antibacterial activity against *Staphylococcus aureus*, *Bacillus subtilis*, and *Escherichia coli* [17,18,19,20]. Of particular interest among them is *I. scariosa*, whose roots and rhizomes contain irigenin, irilon, nigricin and its glycosides, mangiferin and neomangiferin, which have pronounced anti-inflammatory activity and are promising for use in topical forms [19].

The development of soft dosage forms based on plant extracts requires a well-informed choice of polymer base, since the properties of the matrix determine the stability of the drug, its rheological characteristics, the release profile of active ingredients, and the effectiveness of transdermal delivery, as well as ensuring optimal gel consistency, adhesion to the skin, and comfort during application. At the same time, ensuring the quality and safety of the drug is impossible without systematic risk management: in accordance with ICH Q9 and GMP rules, risk analysis must be carried out at all stages of the drug life cycle, and the use of the FMEA [21], regulated by GOST R 51901.12–2007 and GOST R 27.303–2021, allows identifying potential failures of technological processes and minimizing their impact on the stability, safety, and pharmacological efficacy of soft forms.

Thus, the aim of this study is to develop an original formulation of a medicinal gel based on a thick extract of *I. scariosa* with an assessment of its anti-inflammatory activity, which will fill existing gaps in the use of this plant raw material and expand our understanding of its pharmaceutical potential.

## 2. Results and Discussion

### 2.1. Optimization of the Ultrasonic Extraction Process of Thick Extract from I. scariosa Rhizomes

Based on the analysis of literature data, it has been shown that the parameters of ultrasonic extraction, including the composition of the solvent (ethanol:water), processing time, and the ratio of raw material:extractant, significantly affect the yield and composition of biologically active substances from plant material [22,23].

The optimization of the ultrasonic extraction process of biologically active substances from the rhizome of *I. scariosa* began with the selection of the optimal extractant composition. It is known that water, having high polarity, promotes the extraction of a wide range of hydrophilic compounds, but its use may be accompanied by microbiological instability of extracts, increased energy consumption during concentration, and possible hydrolysis of individual components. Ethyl alcohol is a polar solvent that exhibits preservative properties at concentrations above 20%, is technologically safe, and reduces the heat costs of evaporating extracts. The use of water-alcohol mixtures allows the polarity of the extractant to be adjusted, thereby increasing the efficiency of extracting a complex of extractive substances from plant raw materials [24,25].

**Figure 1:**
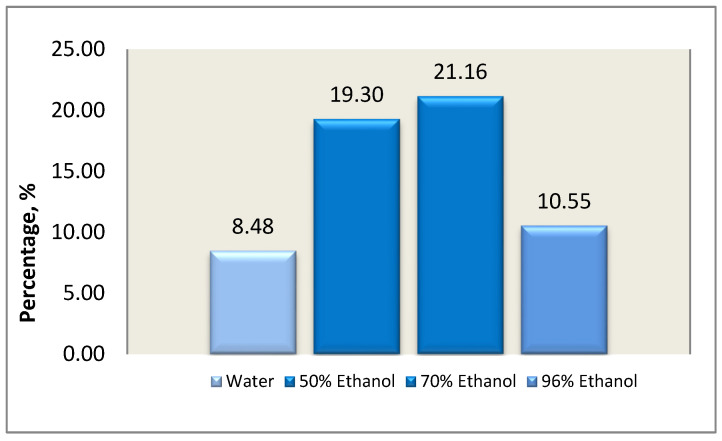
Extraction yield isolated from *I. scariosa* plants during ultrasonic extraction using ethyl alcohol of varying concentrations.

In this study, the extraction of *I. scariosa* rhizomes was carried out using ultrasonic treatment with ethyl alcohol of various concentrations—50%, 70%, and 96%—as well as distilled water. Extraction was carried out using ultrasonic treatment with fixed process parameters: plant material mass—10.0 g, raw material:extractant ratio—1:30 (m/vol), temperature—20–22 °C, duration of one extraction—30 min. Based on the data from the experiment, 70.0% ethyl alcohol was selected as the most optimal solvent (Figure 1).

The yield of extractive substances was statistically significantly dependent on ethanol concentration (*p* < 0.05). The highest yield was obtained using 70% ethanol (21.16 ± 1.21%), which was statistically higher than the yields for water (8.48 ± 3.18%) and 96% ethanol (10.55 ± 0.73%). Although 50% ethanol also provided a high yield (19.30 ± 1.49%), it was slightly lower compared to the 70% solution. Thus, 70% ethanol was found to be the optimal solvent for ultrasonic extraction of biologically active compounds from *I. scariosa* rhizomes.

### 2.2. Phytochemical Profile of I. scariosa: Literature Overview

According to published data, *I. scariosa* is characterized by a high content of phenolic secondary metabolites, among which flavonoids, isoflavones, and xanthone derivatives dominate. The main objects of isolation and structural identification were the rhizomes of the plant, which are considered to be the most biochemically active organs.

Among the flavonoids in *I. scariosa*, apigenin, quercetin-3-glucoside, rutin, hispidulin, and pinocembrin have been described, which possess predominantly antioxidant and anti-inflammatory activity. Isoflavones are represented by irigenin, irisolidone, genistein, tectorigenin, and tectoridin, which are characterized by estrogen-like, cytotoxic, and enzyme-inhibiting properties.

Xanthone derivatives, including iriflophenone, mangiferin, and neomangiferin, complement the plant’s spectrum of biological activity, enhancing its antioxidant and anti-inflammatory potential.

Summary information on the compounds identified in *I. scariosa*, as well as their prevalence among other species of the genus *Iris*, is presented in Table 1.

According to the literature data presented, the dominant groups of compounds in *I. scariosa* are flavonoids and isoflavones, mainly localized in the rhizomes. The frequent detection of isoflavone derivatives indicates their chemotaxonomic significance for the genus *Iris*. The presence of xanthone glycosides, including mangiferin and neomangiferin, indicates the plant’s high antioxidant potential. The detection of similar compounds in other members of the genus confirms the chemical conservatism of secondary metabolism within the genus *Iris*, while quantitative indicators of their content may vary depending on geographical and environmental conditions.

To develop and refine the available literature data, we conducted our own analysis of the phenolic profile of *I. scariosa* extracts.

The study was conducted at Karaganda Medical University (Karaganda, Kazakhstan). Chromatographic analysis revealed six main compounds with retention times of 5.19–17.01 min and [M–H]^−^ ions. Gallic acid, catechin, epicatechin, p-coumaric acid, apigenin-7-O-glucuronide, and rosmarinic acid were identified [1].

### 2.3. Chemical Composition of Essential Oil Determined by GC–MS

Gas chromatography–mass spectrometry (GC–MS) analysis of the essential oil obtained from the rhizomes of *Iris scariosa* identified 33 components, which account for more than 99% of the total ion current (TIC) (Table 2). The analysis was performed under the conditions described in Section 3.2.1.

Retention indices were additionally used to improve the reliability of identification. Estimated RI values (RI (est.)) were calculated based on the retention times of the available n-alkanes (C12–C14) recorded in the chromatogram and were used as indicative parameters. The obtained values were compared with literature values (RI (lit.)). It should be noted that due to the limited number of reference alkanes, the RI values are approximate; however, their agreement with literature data confirms the correctness of the compound identifications [33].

For certain compounds (e.g., 2-undecanone), slightly more pronounced discrepancies are observed between RI (est.) and RI (lit.), which may be attributed to the limited number of reference alkanes and the specific characteristics of the temperature profile used in the analysis. Compounds with poor spectral matches or chemically implausible structures were excluded from the analysis. Components for which complete structural identification could not be achieved are designated as tentative.

**Figure 2:**
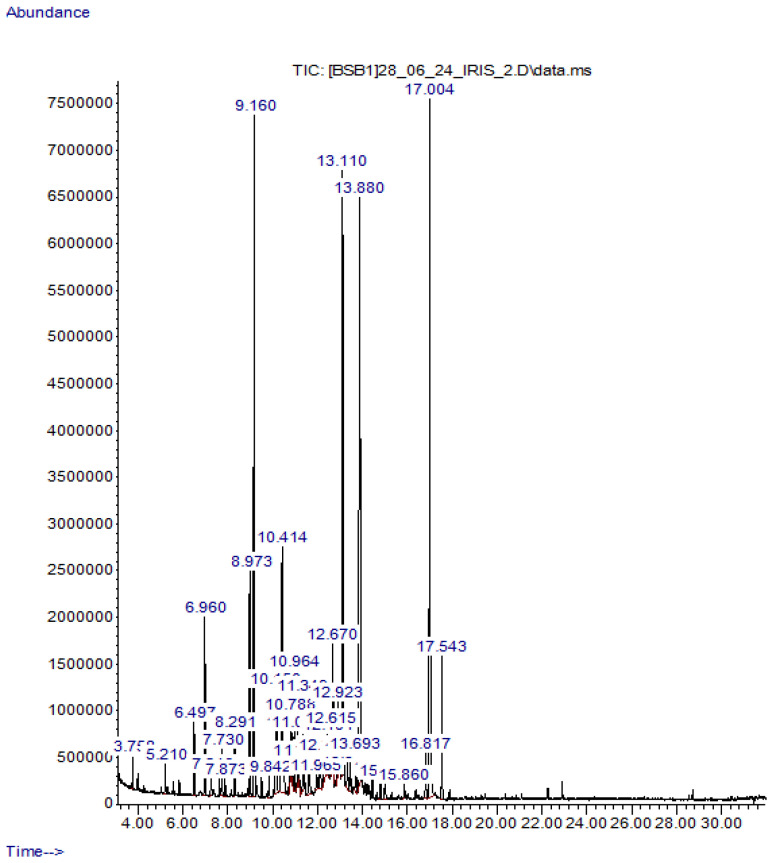
GC–MS chromatogram of the essential oil from the rhizomes of *I. scariosa*. Peaks were identified by comparing mass spectra with the NIST 2017 library using AMDIS software.

The chromatogram of the essential oil (Figure 2) shows a characteristic distribution of major and minor components. The most intense peaks correspond to dodecanoic (lauric) acid, n-decanoic acid, octanoic acid, camphor, and tridecane, which are also the dominant compounds listed in Table 2.

The chemical composition of the essential oil is characterized by a marked predominance of medium-chain saturated fatty acids (C8–C12). The main components are dodecanoic acid (16.38 ± 0.01%), n-decanoic acid (16.11 ± 0.01%), octanoic acid (7.27 ± 0.01%), camphor (13.11 ± 0.75%), and tridecane (9.79 ± 0.01%). The total content of saturated fatty acids (C8–C12) reaches 39.75%, indicating the oil’s pronounced lipophilic nature.

The monoterpene fraction consists primarily of α-pinene, β-pinene, terpinene-4-ol, and camphor. Among these, camphor (RI 1141) is the dominant oxygen-containing monoterpene, indicating a shift in the terpene profile toward oxidized derivatives. The relatively low content of hydrocarbon monoterpenes compared to oxygen-containing derivatives indicates a shift toward more oxidized terpene compounds.

Aliphatic hydrocarbons make up a significant portion of the essential oil and are represented primarily by tridecane (RI 1300), dodecane (RI 1200), and tetradecane (RI 1400), as well as branched and cyclic derivatives such as methyldodecanes and heptylcyclohexane. The presence of these compounds reflects a pronounced hydrocarbon component that forms the oil’s overall lipophilic profile.

Minor components include phenolic and phenylpropanoid compounds, such as eugenol (RI 1356) and 2-methoxy-4-vinylphenol (RI 1310), which, despite their low content, may contribute significantly to the biological activity of the essential oil through synergistic effects.

**Figure 3:**
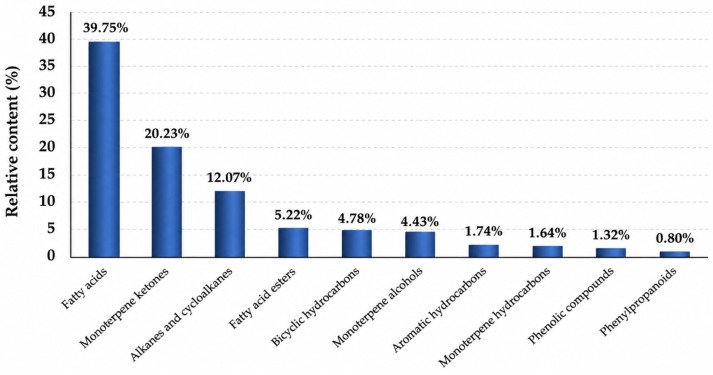
Relative distribution (%) of chemical classes identified in the essential oil of *Iris scariosa* based on GC–MS analysis. Only compounds confirmed by mass spectral matching were included in the classification.

The distribution of compounds by chemical class is shown in Figure 3.

The distribution of chemical classes of compounds (Figure 3) shows that the dominant group is medium-chain fatty acids (39.75%), followed by monoterpene ketones (20.23%), alkanes and cycloalkanes (12.07%), and fatty acid esters (5.22%). In smaller quantities, bicyclic hydrocarbons (4.78%), monoterpene alcohols (4.43%), aromatic hydrocarbons (1.74%), monoterpene hydrocarbons (1.64%), phenolic compounds (1.32%), and phenylpropanoids (0.80%) [34].

The predominance of oxygen-containing monoterpenes, particularly camphor, along with a high fatty acid content, may indicate the oil’s potential biological activity.

A comparison with the literature shows that the obtained chemical profile is consistent with previously described compositions of essential oils from the rhizomes of other species of the genus *Iris*, including *I. carthaliniae*, *I. medwedewii*, and *I. halophila*, which are also characterized by a predominance of medium-chain fatty acids and oxygen-containing compounds [31,35,36].

Medium-chain fatty acids (lauric, decanoic, octanoic) and monoterpenes (α- and β-pinene) are known for their antimicrobial and anti-inflammatory activity, which may determine the pharmacological potential of the essential oil under study [37]. Thus, the essential oil of *Iris scariosa* rhizomes is characterized by a complex lipophilic-terpene profile dominated by medium-chain fatty acids and oxygen-containing monoterpenes, with a significant proportion of aliphatic hydrocarbons also present.

### 2.4. Semi-Quantitative Determination of Polyphenol Content by HPLC

The phenolic profile of the concentrated extract of *I. scariosa* was analyzed by HPLC under the conditions described in Section 3.2.2.

**Figure 4:**
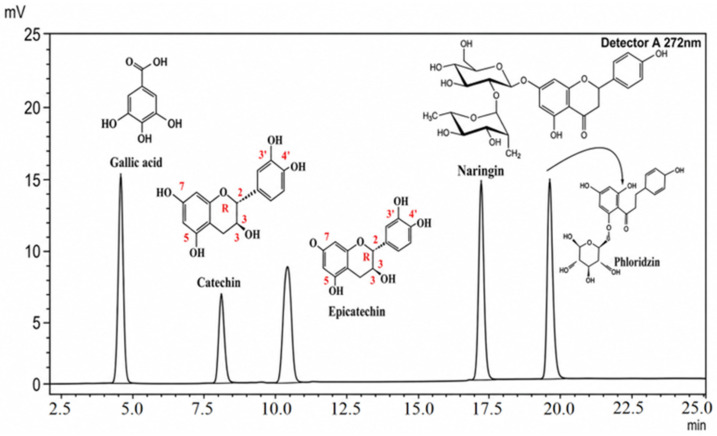
Chromatogram of standards (Gallic acid, Catechin, Epicatechin, Naringin, Phoridzin), UV at a wavelength of 272 nm.

**Figure 5:**
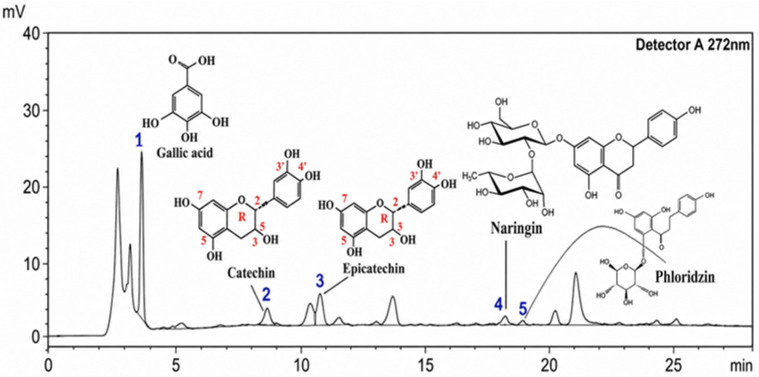
Chromatogram of thick extract *I. scariosa*, rhizomes: UV at a wavelength of 272 nm, gallic acid (1), catechin (2), epicatechin (3), naringin (4), phoridzin (5).

**Figure 6:**
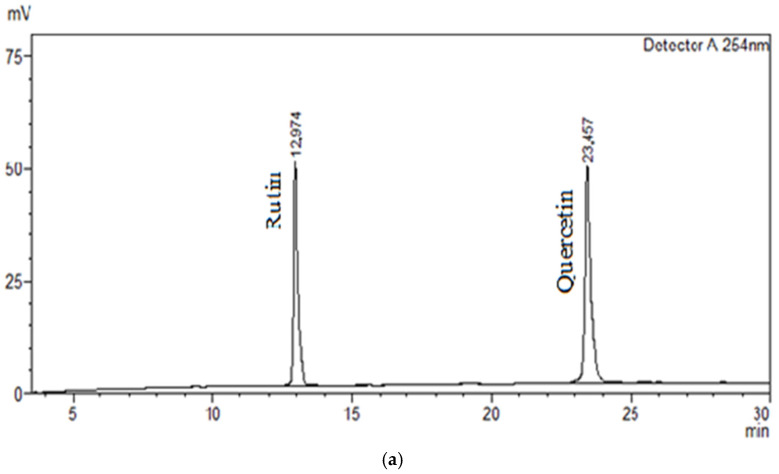

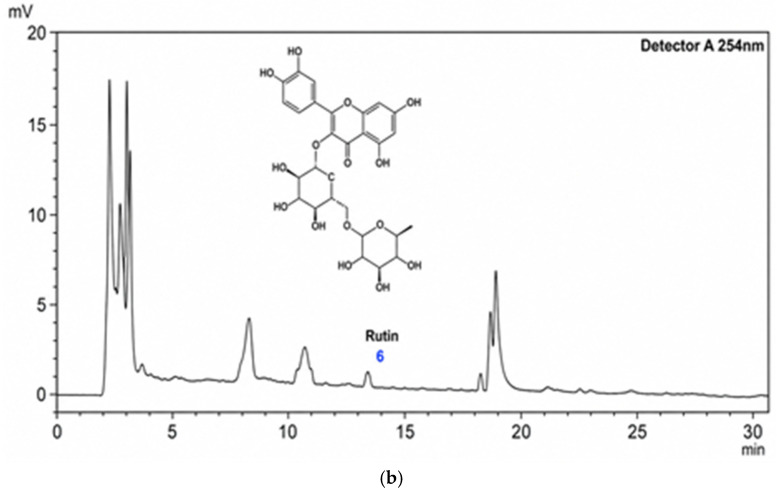
Chromatogram of thick extract *I. scariosa*, rhizomes: UV at a wavelength of 254 nm. (**a**)—Chromatogram of standards (Rutin, Quercetin); (**b**)—Chromatogram of thick extract *I. scariosa*, rhizomes: rutin (6).

The components were separated using an acetonitrile-1% acetic acid gradient system with detection at 272 nm. Compounds were identified by comparing the retention times of the analyzed peaks with those of reference standards (gallic acid, catechin, epicatechin, naringin, phlorizin, rutin, quercetin). The acceptable deviation in retention time was no more than ±0.5 min. Quercetin was not detected in the test sample. Chromatograms of the standard samples and the test extract are shown in Figure 4, Figure 5 and Figure 6.

The limits of detection (LOD) and quantification (LOQ) ranged from 0.50 to 1.07 mg/L and 1.51 to 3.25 mg/L, respectively, indicating that the method was sufficiently sensitive. The lowest LOD and LOQ values were observed for naringin, whereas the highest values were recorded for gallic acid (Table 3).

The precision of the method, assessed by the RSD value for repeated injections (*n* = 3), showed low variability (RSD < 1%), indicating high instrumental repeatability of the analysis [38].

The obtained values (mg/g) should be considered as semi-quantitative estimates based on external calibration and are intended for comparative evaluation rather than absolute quantification.

The total content of the identified phenolic compounds was estimated to be approximately 108.7 mg/g of extract (Table 4). The predominant compounds, based on semi-quantitative evaluation, appear to be epicatechin (49.118 mg/g) and gallic acid (48.077 mg/g), together accounting for approximately 89% of the total estimated content.

Flavan-3-ols (epicatechin and catechin) account for more than half of the total estimated phenolic content based on semi-quantitative analysis (≈52.5%). This class of compounds is characterized by the presence of an ortho-dihydroxyl structure in the B-ring and a hydroxyl group at the C-3 position, which accounts for their high antioxidant activity due to their ability to donate electrons and hydrogen atoms, as well as to stabilize phenoxyl radicals. Compounds **2** and **3**, identified as catechin and epicatechin, have previously been detected in various species of the genus *Iris* (*I. germanica*, *I. schachtii*, *I. pseudacorus*), where they are considered biomarkers of proanthocyanidins and key components of antioxidant activity, as well as compounds associated with pronounced antimicrobial and antitumor activity [27]. In our previous study using HPLC-UV-ESI-MS, catechin and epicatechin were also reported in *I. scariosa* extract. However, direct quantitative comparison should be interpreted with caution due to differences in analytical approaches and calibration procedures [1]. The present semi-quantitative data suggest a significant contribution of flavan-3-ols to the antioxidant potential of the extract and are consistent with literature reports.

Gallic acid accounted for approximately 44.2% of the total estimated phenolic profile, suggesting its predominant contribution within the semi-quantitative profile. Gallic acid contains three hydroxyl groups on the aromatic ring, which are associated with strong antioxidant activity [39]. Its presence in rhizomes has been previously reported for several Iris species, confirming its role as a characteristic phenolic marker of the genus [27].

According to the literature, gallic acid is characterized by a wide range of biological activity, including pronounced antitumor, cardioprotective, and neuroprotective effects, as well as potential in the prevention of neurodegenerative and metabolic diseases. It has been established that its pharmacological properties are largely due to its antioxidant activity, ability to modulate cell proliferation and apoptosis signaling pathways, and anti-inflammatory effects [28,30,31]. It should be noted that in our previous study, gallic acid was also reliably identified by HPLC, which confirms the reproducibility of the data obtained and the stability of the phenolic profile of the species under study [1].

Peaks corresponding to naringin and phlorizin were detected in the *I. scariosa* extract. Their identification is based on matching retention times with reference standards and should be considered tentative, requiring further confirmation by LC–MS/MS. The estimated content of these compounds was relatively low (≤1.4%), but they may contribute to the overall biological activity of the extract. Naringin is known for its antioxidant, anti-inflammatory, and metabolically active properties [40,41], while phlorizin exhibits antidiabetic, antioxidant, and antimicrobial effects [42].

Rutin was detected in a relatively low level of 0.49% of the total phenolic profile of the extract, indicating its minor but qualitatively significant presence. Despite its low quantitative content, rutin is of pharmacological interest due to its well-documented biological effects, including antioxidant, anti-inflammatory, and antimicrobial activity, as well as angioprotective and cardioprotective effects. The mechanisms of its activity are associated with its ability to stabilize cell membranes, inhibit lipid peroxidation, and modulate inflammatory signaling cascades [27]. Rutin was previously detected in the phytochemical profile of *I. scariosa* [26] and *I. schachtii* [26], confirming the characteristic nature of flavonol glycosides for members of the *Iris genus*.

Thus, the rhizomes of *I. scariosa* are characterized by relatively high levels of phenolic compounds based on semi-quantitative analysis, with flavan-3-ols and phenolic acids (primarily gallic acid) representing the predominant groups. The presence of additional flavonoids (rutin, naringin, phlorizin) reflects the complexity of the phenolic composition and suggests potential antioxidant and anti-inflammatory activity of the plant material.

### 2.5. Development and Optimization of a Gel Based on I. scariosa Rhizome Extract

Given the pronounced phenolic profile of the *I. scariosa* rhizome extract, represented mainly by flavan-3-ols and phenolic acids, a hydrophilic gel system based on Carbopol 940 (INCI: Carbomer) was selected for the development of a topical dosage form. This polymer is a cross-linked polyacrylate capable of forming transparent, highly viscous hydrogels with pronounced rheological and bioadhesive properties, making it an optimal matrix for the delivery of polyphenolic compounds in dermatological preparations [43,44].

Carbomers are widely used in soft dosage forms due to their biocompatibility, low toxicity, and ability to provide controlled release of active substances. A stable gel structure forms within a slightly acidic to neutral pH range (≈5.0–6.5), corresponding to the physiological pH of the skin and minimizing the risk of irritation upon topical application [45,46].

#### 2.5.1. Formulation of Model Compositions

To optimize the formulation, five prototype samples (GISU70-1–GISU70-5) were developed by varying the concentrations of carbomer (1.0–3.2% *w*/*w*) [31] and plant extract (2.5–5.0% *w*/*w*). The composition of the formulations is presented in Table 5. The concentration of *I. scariosa* essential oil was kept constant across all samples, allowing evaluation of the effect of the structuring agent on gel formation, rheological behavior, and aggregate stability under constant pharmacological load.

All developed gel bases met the requirements of the State Pharmacopoeia of the Republic of Kazakhstan [10] and the Pharmacopoeia of the Eurasian Economic Union for soft dosage forms for external use [8].

Glycerin (28% *w*/*w*) was included as a humectant and rheological modifier to improve plasticity, prevent drying, and enhance spreadability. This concentration is consistent with commonly used ranges (10–30%) in topical formulations [47].

To incorporate the essential oil into the hydrophilic matrix, the nonionic surfactant Polysorbate 80 (Tween 80) was used at a concentration of 0.3% (*w*/*w*), acting as a dispersing agent to ensure uniform distribution of the lipophilic phase without complete micellar solubilization [48].

The plant extract concentration (2.5–5.0% *w*/*w*) was selected based on literature data for dermatological phytopreparations, ensuring sufficient biological activity without compromising gel structure [49,50].

#### 2.5.2. Gel Production Technology

The optimized preparation method included the following stages:

Polymer swelling—dispersion of Carbopol 940 in purified water under controlled stirring until complete hydration;

Partial neutralization—gradual addition of NaOH solution (10%) to adjust the pH to 5.5–6.0 (typically around 0.2 mL, depending on formulation composition), ensuring the formation of a gel structure;

Preparation of active components—incorporation of the plant extract and predispersion of the essential oil using Tween 80.

Incorporation into gel base—gradual addition under controlled stirring.

#### 2.5.3. Final pH Adjustment and Structure Stabilization

Monitoring included pH control and dosing of the plant extract during gel formation.

It should be noted that the formation of the Carbopol 940 gel structure is governed primarily by the degree of neutralization of carboxylic groups rather than the absolute amount of neutralizing agent added. Although initial aqueous dispersions of Carbopol exhibit an acidic pH (~2.5–3.5), partial neutralization with sodium hydroxide leads to ionization of -COOH groups into -COO^−^, resulting in electrostatic repulsion, expansion of polymer chains, and formation of a three-dimensional gel network.

In the present study, NaOH solution (10%) was added gradually to adjust the pH to 5.5–6.0 (typically around 0.2 mL, depending on formulation composition). This is because complete neutralization of the polymer is not required for gel formation; partial neutralization is sufficient to induce gelation, while the final pH is further influenced by the multicomponent nature of the system.

The aqueous solution of *I. scariosa* extract was found to be slightly acidic (pH ≈ 4.8–5.3), indicating that it does not possess a basic character but contributes to intermolecular interactions within the system.

The transparency of the gel is explained by the use of Tween 80 as a dispersing agent, forming a finely dispersed colloidal system with droplet sizes below the wavelength of visible light. The structured Carbopol network further stabilizes the system by preventing droplet coalescence, even at a low surfactant-to-oil ratio (1:10, *w*/*w*).

#### 2.5.4. Organoleptic Characteristics and Physical Stability

All samples obtained were transparent, homogeneous gels of light brown or brownish color with a characteristic smell of plant raw materials. The organoleptic characteristics and physical stability of the samples were evaluated in three independent replicates (*n* = 3) over a 90-day storage period under various temperature conditions. In all cases, no signs of phase separation or system destabilization were observed (Table 6).

All of the GISU gel formulations developed exhibited stable organoleptic characteristics during storage. The samples varied in color from light brown (GISU70-1 and GISU70-2) to brownish (GISU70-3–GISU70-5), while retaining the characteristic odor of the plant extract.

In terms of appearance, sample GISU70-1 had a more fluid consistency, whereas the other formulations had a gel-like or jelly-like structure with good spreadability. All samples were homogeneous and transparent, with no phase separation observed throughout the storage period. Stability studies conducted at 25 ± 2 °C and 4 ± 2 °C, both for short-term storage (1 day) and long-term storage (90 days), revealed no visible changes in color, odor, or consistency, indicating satisfactory physical stability of the developed gels.

During the cyclic stability test (six temperature cycles), a slight decrease in viscosity was observed for the GISU70-1 sample, whereas the GISU70-2, GISU70-3, and GISU70-4 formulations remained stable without noticeable changes. At the same time, the GISU70-5 sample demonstrated an increase in structural density, which may indicate a possible strengthening of the gel network under stress conditions.

Overall, the results obtained confirm the acceptable organoleptic properties and stability of the developed phytogel formulations, which justifies the feasibility of further pharmaceutical and pharmacological research on them.

#### 2.5.5. pH Values

The results of the pH measurements for the developed gel compositions are presented in Table 7. Measurements were performed in three independent replicates (*n* = 3) after preliminary dilution of the samples, which was necessary to ensure proper contact between the electrode and the semi-solid system and to obtain reproducible results. The results of the pH measurements for the developed gel compositions are presented in Table 7. Measurements were performed in three independent replicates (*n* = 3) after preliminary dilution of the samples, which was necessary to ensure proper contact between the electrode and the semi-solid system and to obtain reproducible results.

During 90 days of storage under controlled temperature conditions, no significant fluctuations in the pH value were observed. At the end of the observation period, the values ranged from 5.61 to 5.89. The changes were insignificant and fell within the limits of acceptable analytical deviations, indicating the stability of the system and the absence of pronounced degradation processes of the active components or structural destabilization of the polymer matrix. A slight increase in pH in some samples (in particular, GISU70-5) may be associated with a higher concentration of carbomer and the characteristics of its partial neutralization, which affects the ionization of the polymer’s carboxyl groups. Despite the noted changes, all pH values obtained remained within a physiologically acceptable range (≈5.0–6.0), which minimizes the risk of skin irritation and does not disrupt the acid-base balance of the epidermal barrier.

Thus, the stability of the pH indicator throughout the entire storage period confirms the correctness of the selected formulation and technological approach, as well as the compatibility of the plant extract, essential oil, and excipients in the gel system.

##### Justification for the Choice of the Optimal Composition

Based on the results of a comprehensive assessment of the technological, organoleptic, and physical-mechanical characteristics, samples GISU70-3 and GISU70-4 were recognized as the most promising.

GISU70-3 (1.5% carbomer; 5.0% extract) is characterized by the formation of a homogeneous, transparent, and plastic gel structure that ensures uniform distribution of active substances and ease of application to the skin. The sample retained its color, odor, and homogeneity for 90 days of storage at various temperatures. The pH values remained within a physiologically acceptable range, confirming the chemical stability of the system and its dermatological safety. This composition provides an optimal balance between structural integrity and softness of the base.

GISU70-4 (2.5% carbomer; 5.0% extract) has a denser and more stable structure, which was manifested in the preservation of the gel’s shape, the absence of liquefaction during temperature fluctuations, and resistance to cyclic loads. During storage, there were no signs of syneresis, phase separation, or changes in organoleptic indicators. A higher content of the cross-linking agent promotes the formation of a more robust three-dimensional polymer network, ensuring the stability of the composition during long-term storage and transportation [50].

#### 2.5.6. Study of the Kinetics of Active Ingredient Release

**Figure 7:**
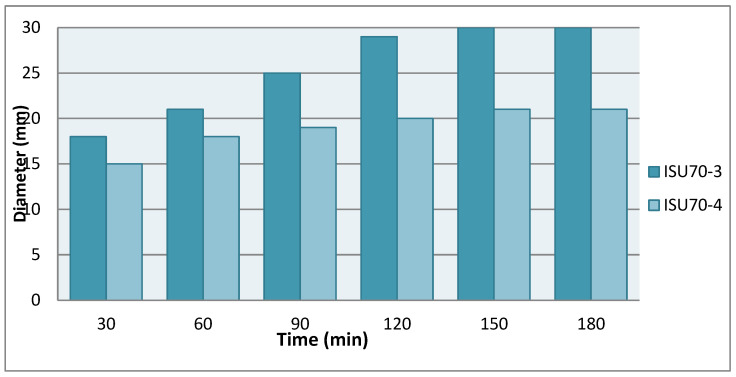
Release kinetics of biologically active compounds from gel formulations (agar diffusion method). Data are presented as mean ± SD (*n* = 3).

The kinetics of release of biologically active substances from gel compositions containing 5% ethanol extract and 3% essential oil of *I. scariosa* were studied by direct diffusion in agar medium. The results are expressed as the mean ± standard deviation (*n* = 3). The data obtained demonstrated a pronounced dependence of the release profile on the nature of the polymer base and its structural characteristics. The most intense and uniform release was observed for the GISU70-3 composition (Figure 7).

The diffusion zone diameter for GISU70-3 increased from 18.0 ± 0.6 mm after 30 min to 30.0 ± 0.6 mm after 180 min, whereas for GISU70-4, the values ranged from 15.0 ± 0.6 mm to 21.0 ± 0.6 mm, respectively. The diffusion curve was characterized by a nearly linear increase in the diameter of the staining zone during the first three hours of the experiment, indicating stable migration of the complex of phenolic compounds in the extract and volatile components of the essential oil into the agar matrix.

A comparative analysis showed that the GISU70-3 formulation exhibited statistically significantly higher diffusion diameters compared to GISU70-4 (*p* < 0.05), indicating more efficient release of the active ingredients.

The obtained profile indicates a negligible influence of diffusion limitations within the gel structure under the experimental conditions and an optimal ratio of hydrophilic and lipophilic components in the system.

The GISU70-3 composition, containing Carbopol-940, Tween-80, purified water, and glycerin, probably forms a balanced three-dimensional network with a sufficient degree of hydration. The presence of the nonionic solubilizer Tween 80 promotes a more uniform distribution of the essential oil in the hydrophilic phase of the gel, which facilitates its diffusion and enhances the efficiency of release. In contrast, the GISU70-4 formulation exhibited a less pronounced increase in the diffusion zone, which may be attributed to the denser structure of the polymer matrix, limiting the mobility of both the phenolic components of the extract and the terpenoid compounds of the essential oil.

Thus, it has been established that the release of the total complex of biologically active compounds from gel systems is determined by the structural characteristics of the matrix, the degree of polymer hydration, and the presence of surfactants. Among the samples studied, the GISU70-3 formulation demonstrated the most favorable release profile and can be considered optimal from a biopharmaceutical standpoint.

#### 2.5.7. Rheological Properties of Developed Gels

The flow curves obtained for the GISU70-3 and GISU70-4 samples demonstrate pronounced non-Newtonian behavior. The relationship τ = f($\dot{\gamma }$) is nonlinear, indicating a pseudoplastic flow type characteristic of structured gel systems.

**Figure 8:**
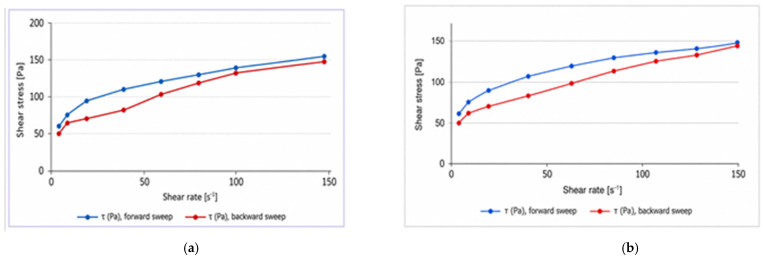
Rheograms of the flow of gel compositions GISU70-3 (**a**) and GISU70-4 (**b**) at 20 °C (ascending and descending branches of the curves τ = f($\dot{\gamma }$)).

For sample GISU70-3 (Figure 8a), a distinct hysteresis loop is observed between the ascending and descending branches of the curve, indicating a thixotropic flow behavior. The presence of hysteresis indicates partial disruption of the gel’s spatial structure as the shear rate increases and its gradual recovery as the mechanical load decreases. Such behavior is characteristic of structured polymer gels with a well-developed three-dimensional network.

A similar flow behavior was observed for the GISU70-4 sample. As shown in Figure 8b, the curve of shear stress versus shear rate exhibits a nonlinear character, confirming the pseudoplastic flow behavior of the system. A hysteresis loop is observed between the ascending and descending branches, indicating the presence of thixotropic properties.

**Figure 9:**
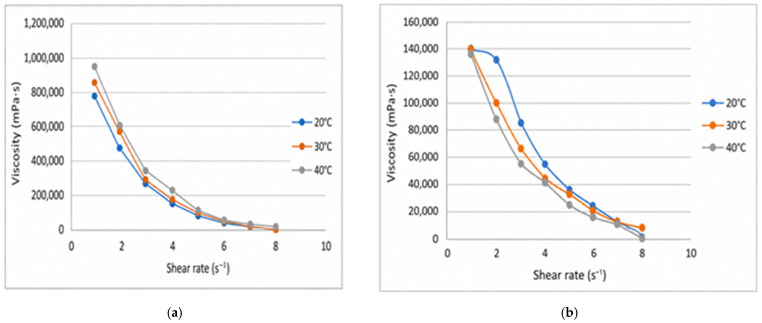
Temperature dependence of the rheological parameters of the GISU70-4 (**a**) and GISU70-3 (**b**) gel compositions: effect of temperature (20–40 °C) on the flow curves τ = f($\dot{\gamma }$).

The temperature dependence of the rheological parameters is shown in Figure 9a for the GISU70-4 sample and in Figure 9b for the GISU70-3 sample. As can be seen in Figure 9a, an increase in temperature from 20 to 40 °C leads to a sequential decrease in shear stress values at constant strain rates. The most pronounced differences are observed in the low shear rate range, indicating the temperature sensitivity of the gel’s spatial structure.

As the temperature increases, the degree of intermolecular interactions in the gel matrix decreases, which manifests as a reduction in the system’s resistance to external mechanical stress.

A similar trend is observed for the GISU70-3 sample (Figure 9b), although the temperature effect is less pronounced. The difference between the curves at 20 and 40 °C in the high shear rate region becomes less noticeable, indicating partial structural breakdown under mechanical loading regardless of the temperature factor.

Thus, for both samples, a temperature dependence of the rheological parameters has been established, in which an increase in temperature is accompanied by a decrease in the structural strength of the system. At the same time, the GISU70-4 sample demonstrates more pronounced temperature sensitivity compared to GISU70-3, which may be associated with the peculiarities of the compositional composition and the nature of structure-forming interactions in the gel system.

A comparative analysis of the temperature rheograms (Figure 9a,b) showed that the GISU70-3 sample exhibits greater thermal stability, as the change in shear stress upon increasing the temperature from 20 to 40 °C is less pronounced compared to GISU70-4.

### 2.6. Antioxidant Activity of the Ethanol Extract and Gel of I. scariosa

The chemiluminescence parameters of spontaneous light and total light in model systems for the generation of reactive oxygen species (ROS, Model I) and lipid peroxidation (LPO, Model II) are presented in Table 8.

*Model of reactive oxygen species (ROS) generation.*

In the control system, a high light integral of 29.4 (27.5–30.1) units and spontaneous luminescence of 1.4 (1.3–1.5) units were recorded, reflecting intense ROS formation.

IS-70-U significantly reduced the light sum to 14.3 (12.7–16.1) units (* *p* ≤ 0.05 compared to the control).

The gel form GISU70-3 demonstrated a comparable reduction—13.1 (10.5–14.3) units (* *p* ≤ 0.05). Spontaneous luminance decreased to 0.9 (0.8–1.0) units for the extract and 0.8 (0.7–1.1) units for the gel.

Thus, the incorporation of a 70% ethanol extract and essential oil of *I. scariosa* into the gel matrix did not result in a loss of antioxidant activity in the ROS generation system. The median light-induced sum values for the gel were comparable to those for the original extract.

Ascorbic acid reduced the light sum to 4.5 (4.3–4.8) units (* *p* ≤ 0.05), demonstrating a more pronounced effect compared to all tested samples (*p* < 0.05), which corresponds to its status as a reference water-soluble antioxidant.

*Model of lipid peroxidation.*

In the POL model, control was characterized by a light sum of 24.7 (23.4–27.8) units and spontaneous luminescence of 1.1 (1.0–1.1) units.

IS-70-U produced a statistically significant reduction in the light sum to 20.1 (17.8–21.3) units (* *p* ≤ 0.05), while simultaneously reducing spontaneous luminescence to 0.9 (0.7–1.0) units. This indicates moderate inhibition of lipoperoxidation processes.

In contrast to the extract, GISU70-3 exhibited a luminance of 23.2 (20.7–25.8) units, which was not statistically different from the control (*p* > 0.05). Spontaneous luminance values were 1.3 (1.0–1.3) units. Thus, in this model, no pronounced suppression of LPO by the gel form was detected.

Ascorbic acid reduced the luminance to 5.6 (5.4–5.8) units (* *p* ≤ 0.05), significantly surpassing the activity of the studied herbal preparations (*p* < 0.05).

The results obtained indicate that the antioxidant activity of the 70% ethanol extract of *I. scariosa* is most pronounced in the ROS generation system, whereas in the LPO model the effect is moderate. The gel formulation containing the extract and essential oil of *I. scariosa* retains antioxidant activity in the ROS system; however, it does not demonstrate statistically significant inhibition in the lipid model.

Differences in activity between the two models may be attributed to the specific phase distribution of the components, the varying solubility of phenolic and terpenoid compounds, as well as the specific nature of radical processes in aqueous and lipidic media. The presented data indicate the superior efficacy of the studied herbal preparations in systems simulating the generation of reactive oxygen species.

The results of this study are consistent with scientific research indicating the presence of phenolic compounds and bioflavonoids with antioxidant activity in species of the genus *Iris*. A recent review noted that extracts of Iris species contain high levels of phenolic acids, flavonoids, and other polyphenolic metabolites, which serve as biochemical markers of antioxidant activity in vitro and in vivo and may also act as potential chemoprotective agents against oxidative stress and related diseases [27,31].

### 2.7. Anti-Inflammatory Activity of GISU70-3 and GISU70-4 Gels in a Formalin-Induced Paw Edema Model in Mice

The anti-inflammatory activity of the developed phytogels GISU70-3 and GISU70-4 was investigated in an experimental model of formalin -induced paw edema in mice, which is considered a reproducible and pathogenetically relevant model of inflammation that reflects the characteristics of inflammatory processes typical of arthritis-like conditions in humans [51]. The efficacy of the tested samples was compared with that of the reference nonsteroidal anti-inflammatory drug—diclofenac sodium (1%)—under in vivo conditions.

The animals were randomized into four experimental groups (*n* = 6 in each): a control group (formalin without treatment), a group receiving topical application of GISU70-3 gel, a group receiving topical application of GISU70-4 gel, and a comparison group receiving diclofenac sodium. The test preparations were applied topically after induction of inflammatory edema.

**Figure 10:**
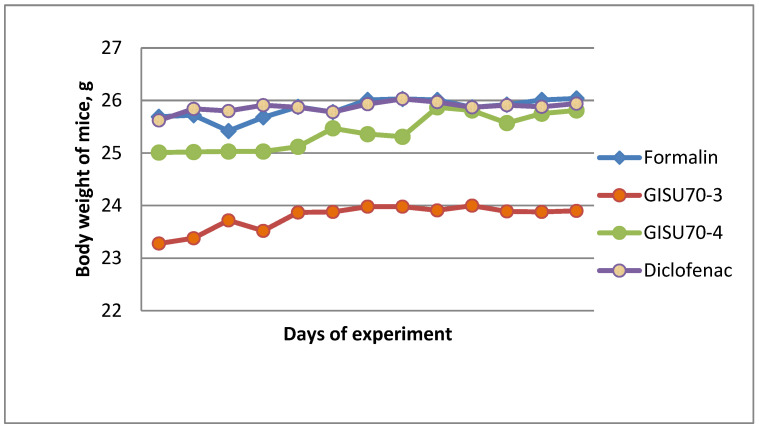
Changes in body weight in mice following subaponeurotic injection of formalin.

Over the 12-day experimental period, the animals’ body weight and the dimensions of the edematous hind paw were recorded daily, allowing for an assessment of the dynamics of the inflammatory process and the possible systemic effects of the test preparations. It was found that the body weight of the animals in all groups remained stable throughout the entire observation period; no statistically significant differences between the groups were detected (*p* > 0.05). The minor fluctuations in the parameters observed were within the range of physiological variability (Figure 10). No animal deaths or clinically significant behavioral changes were recorded during the experiment.

Following formalin injection, the animals developed marked local inflammation characterized by tissue edema and hyperemia. The inflammatory response peaked by the second day of the experiment. Behavioral observations indicated a reduction in weight-bearing on the affected limb; the animals primarily moved on three legs, suggesting the development of inflammatory hyperalgesia.

**Figure 11:**
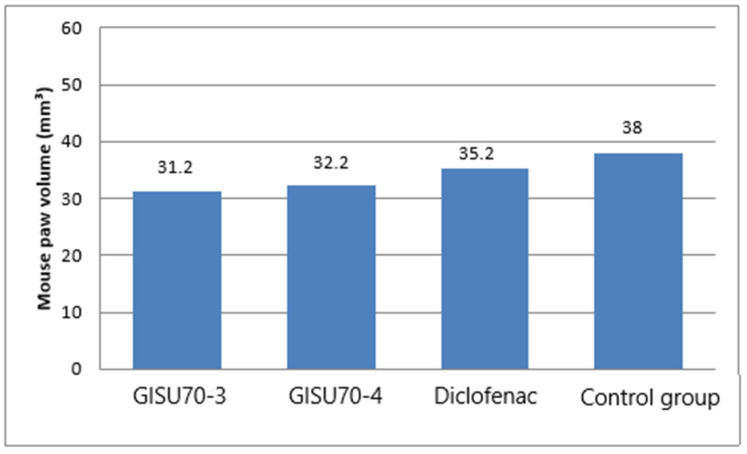
Change in paw volume (ΔV = V − V_0_) between the first (V_0_) and last (V) days of the experiment.

The graph shown in Figure 11 illustrates the difference in paw volume in mice on the first and last days of the experiment.

The difference in paw volume (ΔV = V_12_ − V_1_) showed that formalin injection caused a marked increase in paw volume: ΔV was 38.0 ± 0.65 mm^3^ (*n* = 6), confirming the successful induction of an inflammatory response. The application of 1% diclofenac led to a reduction in edema compared to the formalin group, with ΔV = 35.2 ± 1.35 mm^3^, demonstrating a noticeable anti-inflammatory effect. Topical application of GISU70-3 gel (5.0%) resulted in a statistically significant reduction in paw volume (ΔV = 31.2 ± 0.49 mm^3^) compared to the untreated formalin group (ΔV = 38.0 ± 0.65 mm^3^). The anti-inflammatory effect of GISU70-4 gel (5.0%) was comparable to that of 1% diclofenac (ΔV = 32.2 ± 0.61 mm^3^ and 35.2 ± 1.35 mm^3^, respectively). Overall, the tested samples demonstrated moderate anti-inflammatory activity under conditions of formalin-induced inflammation.

Additionally, the diameter of paw edema was assessed at 0, 4, and 24 h after formalin injection (Table 9). In the control group, a significant increase in paw diameter was observed after 4 h, followed by a partial decrease after 24 h. The application of the phytogels GISU70-3 and GISU70-4 resulted in a significant reduction in paw diameter compared to the control at the corresponding time points (*p* < 0.05). In terms of the severity of the effect, the gels under study were comparable to diclofenac sodium, especially after 24 h, which indicates pronounced anti-inflammatory activity and a sustained therapeutic effect.

Thus, topical application of GISU70-3 and GISU70-4 gels exhibits statistically significant anti-inflammatory activity in a formalin-induced inflammation model in mice, confirming the promise of further pharmacological investigation of biologically active compounds from the plant *Iris scariosa* as potential herbal anti-inflammatory agents.

## 3. Materials and Methods

### 3.1. Collection, Identification of Plants, and Preparation of Extracts

**Figure 12:**
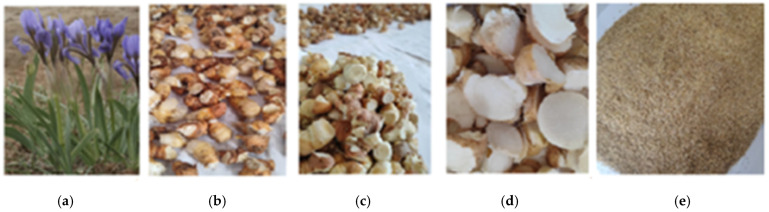
*I. scariosa* during the flowering stage and successive stages of rhizome preparation for analysis: freshly collected rhizomes, cleaned and sliced material, and finely ground powder. The size of rhizome fragments after cutting was approximately 1–2 cm ((**a**)—flowering plants of *I. scariosa*; (**b**)—freshly collected rhizomes; (**c**)—sliced rhizomes; (**d**)—fragments prepared for drying; (**e**)—finely ground rhizome powder for analysis).

The rhizomes of *I. scariosa* were collected during the active growing season and flowering period, on 23–24 April 2024, in the Karaagash Forestry of the Zhanaarka District, Ulytau Region, Republic of Kazakhstan (approximately 48°54′52″ N, 70°54′57″ E) at an altitude of 330–350 m above sea level. The plant material was identified at the Department of Botany of the E.A. Buketov Karaganda National University. The herbarium sample was deposited in the Herbarium of the Department under number QAR12486, which ensures the reproducibility of the study and the possibility of subsequent verification of botanical identification. After collection, the raw materials were thoroughly cleaned of soil and damaged areas, washed with water, and dried at room temperature. For subsequent extractions, the rhizomes were cut into pieces (~1–2 cm), ground, and sieved to obtain a homogeneous powder. Figure 12 illustrates all the key stages of plant material preparation: from the original plant to the finished powder used for extraction.

Ultrasonic extraction was used to extract biologically active substances from the underground organs of *I. scariosa*. A sample of dried raw material (10.0 g) was poured with 300 mL of solvent (distilled water or ethanol:water mixtures) in a ratio of 1:10 (volume/volume). Extraction was carried out using an Ultrasonic Cleaner Sonic-3 device (Stegler, Beijing, China) at a frequency of 40 kHz, a temperature of 20–22 °C, and a processing time of 30 min. After decanting, the process was repeated under the same conditions. The resulting extracts had different concentrations of ethanol (50%, 70%, 96%) and distilled water.

The combined solutions were filtered through a paper filter and evaporated on a rotary evaporator at 50 °C, then the residual solvent was removed in a water bath at 60 °C. The resulting thick extracts were dark brown masses with a specific odor; the yield of extractive substances was 8.5–21.2% depending on the solvent composition.

### 3.2. Chemical Characteristics of the I. scariosa Extract

#### 3.2.1. Essential Oil Extraction and GC-MS Analysis

Essential oil from the rhizomes of *Iris scariosa* was extracted by hydrodistillation using a Clevenger-type apparatus. For this purpose, 20 g of prepared plant material was mixed with 200 mL of distilled water and distilled for 3 h. The yield of essential oil was calculated as a percentage (*w*/*w*) relative to the dry weight of the plant material. The obtained essential oil was stored in sealed amber vials at −20 °C until GC–MS analysis [52]. GC–MS analysis was performed using an Agilent 7890A gas chromatograph (Santa Clara, CA, USA) coupled with an Agilent 5975C mass selective detector. Separation was achieved on an HP-5MS capillary column (30 m × 0.25 mm, film thickness 0.25 μm). The oven temperature program was as follows: initial temperature 70 °C (2 min), increased to 270 °C at a rate of 20 °C/min, and held at 270 °C for 30 min. Helium was used as the carrier gas at a constant flow rate of 2 mL/min. The injector temperature was 250 °C and the detector temperature was 230 °C [53]. Mass spectra were recorded under electron impact (EI) ionization at 70 eV. The ion source temperature was 280 °C and the mass scan range was *m*/*z* 40–400.

The sample was analyzed without prior derivatization. Prior to analysis, the essential oil was diluted in n-hexane (1:100, *v*/*v*). One microliter of the solution was injected in split mode (split ratio 1:20).

Compounds were identified by comparing their mass spectra with those in the NIST 2017 library using AMDIS software (version 2.71). The relative content of components (%) was calculated by peak area normalization without applying correction factors.

Retention indices (RI) were used to support compound identification. RI values were estimated based on the retention times of n-alkanes detected in the chromatogram under identical analytical conditions. Due to the limited number of reference alkanes (C12–C14), the calculated RI values were considered approximate and used as indicative parameters. The obtained RI values were compared with literature data reported for non-polar columns (HP-5MS type) from [33].

#### 3.2.2. Semi-Quantitative Determination of Components by HPLC

An analysis of the chemical composition of the phenolic compounds in the extract of *I. scariosa* was conducted at the Research Institute of Natural Products and Technologies (Almaty, Kazakhstan).

The phenolic profile of a concentrated ethanol extract obtained from the rhizomes of *I. scariosa* was analyzed by high-performance liquid chromatography (HPLC). The analysis was performed on a Shimadzu LC-40 series chromatographic system equipped with a UV detector and controlled by LabSolutions software (version 5.1). A concentrated extract obtained using 70% ethanol, without additional fractionation, was used for the analysis.

Sample preparation consisted of dissolving 1 mg of the concentrated extract in 1 mL of methanol. A 10 μL volume of the resulting solution was injected into the chromatographic system. The autosampler temperature was maintained at 40 °C.

The separation of components was carried out on a reversed-phase C18 column (250 × 4.6 mm, sorbent particle size 5 μm). Solution A (1% acetic acid in water) and solution B (acetonitrile) were used as the mobile phase. Elution was performed in gradient mode, starting with 10% solvent B, followed by a linear increase in its content to 90% over 55 min. The eluent flow rate was 0.7–1.0 mL/min. Detection was performed at a wavelength of 272 nm, selected based on preliminary UV scanning that revealed the maximum absorption of phenolic compounds.

Compounds were identified by comparing the retention times of the analyzed peaks with those of standard samples (gallic acid, catechin, epicatechin, naringin, phlorizin, rutin, and quercetin) recorded under identical chromatographic conditions. The acceptable deviation in retention time was no more than ±0.5 min [54,55].

Semi-quantitative determination of the content of individual compounds was performed using calibration curves constructed by the external standard method. Calibration curves were plotted over a concentration range of 6.125–67.375 mg/L, using three replicates for each data point. In all cases, high linearity was observed (R^2^ = 0.9993–0.9995).

Concentrations were converted from mg/L to mg/g of extract using the following formula:

$$C_{mg/g}=\frac{C_{mg/L}\cdot V}{m}$$

where *C*_mg/L_ is the concentration in solution, V = 0.001 L is the volume of the solution, and m = 0.001 g is the mass of the extract. Under these conditions, the conversion of concentration (mg/L) to content (mg/g) was performed taking into account the ratio of extract mass to solvent volume (1 mg/1 mL), which under these experimental conditions leads to a coincidence of the numerical values of the concentrations.

The limit of detection (LOD) and the limit of quantification (LOQ) were calculated in accordance with the recommendations of ICH Q2(R1) using the following formulas:

$$LOD=\frac{3.3\cdot \sigma }{S}\;LOQ=\frac{10\cdot \sigma }{S}$$

where σ is the standard deviation of the response, and *S* is the slope of the calibration curve.

The precision of the method was assessed by the relative standard deviation (RSD, %) based on three consecutive injections of the same solution (*n* = 3), which reflects the instrumental repeatability of the analysis. It should be noted that the presented approach is semi-quantitative in nature, since a full validation of the method (including an assessment of recovery, accuracy, and reproducibility of sample preparation) was not performed within the scope of this study. Additional sources of uncertainty may include matrix effects and compound identification based primarily on retention time. The determination of rutin was performed on an estimated basis due to the absence of a complete calibration curve.

### 3.3. Development of a Gel Based on I. scariosa Extract

The pharmaceutical substance in the gel is an ethanol extract of *I. scariosa*, which has high anti-inflammatory activity. To develop an optimal formulation, several model gel compositions were prepared and subsequently evaluated for their pharmaceutical-technological characteristics and biopharmaceutical properties.

As part of the study, five experimental gel samples based on Carbopol 940 (INCI: Carbomer) were prepared.

#### 3.3.1. Organoleptic Characteristics and Appearance

The developed gel compositions were evaluated for long-term physicochemical stability. The analysis included the determination of organoleptic characteristics (color, odor, appearance), pH, electrical conductivity, and rheological parameters 24 h after preparation (baseline) and after 90 days of storage [56]. The samples were stored in sealed polymer containers at 4 ± 2 °C (refrigerated storage conditions) and 25 ± 2 °C (controlled storage conditions) in accordance with current pharmacopoeial recommendations for the stability assessment of soft dosage forms for external use.

Additionally, a freeze–thaw stress test was performed: the samples were kept at room temperature for 48 h, then placed in a freezer (−8…−2 °C). This cycle was repeated six times. At the end of the test, the appearance, homogeneity and presence of phase separation were evaluated.

#### 3.3.2. Determination of pH Values

The pH of the gel compositions was determined using a HANNA HI 2020-02 Edge digital pH meter (Smithfield, RI, USA). For analysis, 1.0 g of gel was dispersed in 100.0 mL of distilled water and left at room temperature for 2 h to allow equilibrium to be established. Preliminary dilution of the samples ensured proper contact between the electrode and the semi-solid system and yielded stable, reproducible results; this approach is standard practice in the analysis of semi-solid dosage forms.

Measurements were performed in three independent replicates for each sample, followed by calculation of the mean value. Before starting the measurements, the pH meter was calibrated using standard buffer solutions (pH 4.0, 7.0, and 10.0).

Neutralization was performed partially using a NaOH solution, primarily to adjust the system’s pH to a physiologically acceptable range (5.5–6.5) and to initiate the formation of a gel structure. The amount of neutralizing agent was selected empirically and did not imply complete neutralization of the polymer’s carboxyl groups. The obtained pH values reflect the effective pH of the system’s aqueous phase.

#### 3.3.3. Study of Release Kinetics

To prepare the agar base, 2.00 g of bacteriological agar was placed in a heat-resistant conical flask with a capacity of 250 mL and 100 mL of purified water was added. The mixture was heated in a boiling water bath with constant stirring until the polymer was completely dissolved (approximately 10–15 min) to obtain a transparent homogeneous solution. Ten milliliters of a 0.1% solution of methylene blue was added to the hot solution and stirred until the dye was evenly distributed. After that, the liquid agar was immediately poured into Petri dishes in equal volumes, forming a layer of uniform thickness. Solidification was carried out by cooling until a dense gel structure was formed. After gel formation was complete, cylindrical wells with a diameter of 8.5 mm were formed in the agar plate. Each well was filled with 0.25 g of the test gel containing essential oil in various base variants. Incubation was carried out in a thermostat at a temperature of 36.6 ± 0.2 °C for 3 h. The release of the active component was assessed by the formation of a colored zone around the well, resulting from diffusion. All experiments were conducted in three independent replicates (*n* = 3). Results are expressed as the mean ± standard deviation. The method was used as a comparative test to evaluate the effect of the base composition on the rate of release of the active substance from a semi-solid dosage form [37].

#### 3.3.4. Rheological Characteristics

The rheological properties of gel compositions based on *I. scariosa* extract and essential oil were studied using a Visco Star Plus L rotational rheometer (SP Tech Co., Ltd., Cheongju-si, Republic of Korea) at Karaganda Medical University. Measurements were performed in controlled shear rate mode.

The samples were pre-thermostated in the measuring cell for 30 min until thermodynamic equilibrium was reached. The temperature was maintained at 20, 30, and 40 °C with an accuracy of ±0.1 °C using the device’s built-in thermostating system. Rheological measurements were performed in the shear rate range of 5–150 s^−1^. The protocol included a sequential increase in shear rate (ascending branch) and then its decrease (descending branch), which allowed us to evaluate the structural stability of the system and the presence of thixotropic effects.

The dependence of the tangential shear stress (τ, Pa) on the shear rate ($\dot{\gamma }$, s^−1^) was recorded, and τ = f($\dot{\gamma }$) flow curves were constructed based on the experimental data. Additionally, the dependence of the effective viscosity on the shear rate was analyzed. All measurements were performed in three parallel replicates; the average values were used in the calculations.

The methodological approach complies with the generally accepted principles of rheological analysis of structured dispersed systems described in the classic manual [10].

### 3.4. Antioxidant Activity (Chemiluminescence Method)

The antioxidant activity of the herbal preparations under study was evaluated using the luminol-dependent chemiluminescence method in two experimental models that reproduce the processes of active oxygen species (AOS) generation and lipid peroxidation (LPO). The method is based on the registration of a light signal arising from the oxidation of luminol by active radical forms of oxygen. The intensity of the luminescence reflects the rate of free radical reactions, and its decrease in the presence of the studied samples is interpreted as a manifestation of antioxidant activity. The measurements were performed on a chemiluminometer “HLM-003”. The luminescence was recorded for 5 min, followed by the calculation of the integral light sum (conditional units).

Each reaction system contained 1 mL of the test solution for a total volume of 20 mL. All experiments were performed in six independent replicates (*n* = 6). Ascorbic acid was used as a positive control.

Model I—active oxygen species generation system. The reaction system included phosphate buffer (pH 7.45), luminol (final concentration 10^−5^ M), and Fe^2+^ ions as the reaction initiator. The buffer was prepared by dissolving KH_2_PO_4_ (2.72 g), KCl (7.82 g), and sodium citrate (1.5 g; C_6_H_8_O_7_Na_3_·5.5H_2_O) in 1 L of distilled water, followed by pH adjustment to 7.45 with a saturated KOH solution. The formation of reactive oxygen species was initiated by adding 1 mL of FeSO_4_ solution (50 mM).

Fe^2+^ ions in the presence of dissolved oxygen initiate reactions similar to the Fenton reaction, accompanied by the formation of highly reactive hydroxyl radicals. The concentrations of the reagents were selected experimentally to ensure a stable and reproducible chemiluminescence signal.

Luminol (5-amino-2,3-dihydro-1,4-phthalazinedione) undergoes oxidation to an electronically excited state; the return to the ground state is accompanied by the emission of a photon, which is registered by the device. A decrease in the light sum in the presence of the samples under study indicated their ability to interact with active forms of oxygen, chelate transition metal ions, and inhibit free radical processes.

Model II—lipid peroxidation. To simulate lipid peroxidation, a lipoprotein emulsion prepared from chicken egg yolk was used. The yolk was mixed with phosphate buffer in a 1:5 ratio and homogenized until a homogeneous suspension was obtained. Peroxidation of unsaturated fatty acids was initiated by adding 1 mL of FeSO_4_ solution (50 mM). The development of chain reactions was accompanied by the accumulation of peroxyl radicals, which was reflected in an increase in the intensity of chemiluminescence. The decrease in the integral light sum in the presence of phytopreparations was interpreted as inhibition of lipid peroxidation processes [57]. Comparison preparations: Diclofenac sodium (Grotex LLC, St. Petersburg, Russia), Ascorbic acid (Shandong Xinhua Pharmaceutical Co., Ltd., Zibo, China), Unrefined filtered sunflower oil (Sigma LLC, Moscow, Russia), Ethyl alcohol for the preparation of dosage forms 95% (Konstanta-Farm M LLC, Moscow, Russia).

### 3.5. In Vivo Study of the Anti-Inflammatory Activity of Gels

The anti-inflammatory activity of gel compositions containing plant substances from *I. scariosa* rhizomes was evaluated using a model of formalin-induced inflammatory edema of the hind paw of mice in accordance with generally accepted preclinical recommendations [58]. The experiment used male white laboratory mice aged 2 months with a body weight of 23–26 g. The animals were housed under standard vivarium conditions (temperature 22 ± 2 °C, relative humidity 50–60%, 12-h light cycle) with free access to food and water. The animals were randomized into four groups (6 individuals in each): control group—formalin-induced edema without treatment;

comparison group—1% diclofenac gel;
experimental group 1—GISU70-3 gel (5%);
experimental group 2—GISU70-4 gel (5%).

Inflammation was modeled by subplantar injection of 20 μL of a 3.75% formalin solution into the right hind paw. One hour after induction of inflammation, 40 mg of the test gel or diclofenac gel was applied to the area of edema, followed by gentle rubbing until complete absorption. The treatment was performed daily for 12 days.

Morphometric assessment of inflammation was performed by measuring the length, width, and thickness of the paw with a digital caliper (ASIMETO, Weißbach, Germany) at a fixed time of day. The volume of the paw was calculated using the formula:

V = ½ × (L × W × H)

where L is the length, W is the width, and H is the thickness of the paw.

The efficacy of the anti-inflammatory effect was assessed based on the reduction in paw edema volume compared to the control group. The body weight of the animals was recorded daily throughout the entire experimental period to assess the tolerability of the gels. Results were evaluated with minimal subjective influence from the investigator (whenever possible, using a blinded assessment). All procedures were performed in accordance with the principles of minimizing pain and stress in animals; humane endpoints were applied in accordance with ethical requirements. To minimize pain during inflammation modeling, gentle experimental conditions were used; additional anesthetics were not used due to the specific nature of the model.

### 3.6. Ethical Approval

The study was conducted in accordance with international principles of the humane treatment of laboratory animals and OECD guidelines. The study protocol was approved by the Bioethics Committee of the Karaganda Medical University (Protocol No. 15 dated 2 September 2025, registration number 153 dated 28 August 2025). The experiment was conducted in compliance with ARRIVE guidelines and current regulatory requirements for the care and use of laboratory animals. All procedures were performed with the aim of minimizing animal suffering and reducing the number of animals used.

### 3.7. Statistical Analysis

Statistical processing of the results was performed using the Statistica 10.0 software package (StatSoft Inc., Tulsa, OK, USA). The normality of the data distribution was checked using the Shapiro–Wilk criterion. Due to the deviation of the distribution from normal, nonparametric analysis methods were used. The results are presented as the median and interquartile range (25–75%). The Kruskal–Wallis and Mann–Whitney criteria were used to compare independent samples, and the Friedman criterion was used to analyze repeated measurements. Differences were considered statistically significant at *p* ≤ 0.05.

## 4. Conclusions

The study found that 70% ethanol yields the highest extraction yield (21.16 ± 1.21%) and is the optimal solvent for the ultrasonic extraction of bioactive compounds from the rhizomes of *Iris scariosa*.

Phytochemical analysis revealed that the extract is characterized by a high content of phenolic compounds, dominated by epicatechin (49.12 mg/g) and gallic acid (48.08 mg/g). Catechin, naringin, phlorizin, and rutin were also identified. Flavan-3-ols account for more than 50% of the total phenolic profile, with gallic acid constituting 44.2%. This study is the first to identify naringin and phlorizin in species of the genus *Iris*. The data obtained indicate that the extract possesses significant antioxidant potential.

Analysis of the essential oil revealed a predominance of lipophilic components, including medium-chain saturated fatty acids and terpenoid compounds, which is consistent with the results of GC-MS analysis.

Based on the concentrated extract and essential oil obtained from the rhizomes of *I. scariosa*, a gel dosage form was developed using Carbopol 940 (INCI: Carbomer). Glycerin (28% *w*/*w*) was used as a humectant, and the nonionic solubilizer Polysorbate 80 (Tween 80, 0.3% *w*/*w*) was used to disperse the essential oil. The pH values of all samples were in the range of 5.5–6.0, which corresponds to the physiological pH of the skin.

Five model formulations (GISU70-1–GISU70-5) were developed, varying the concentrations of carbomer and extract. All gels were transparent, homogeneous systems of light brown color with a characteristic odor. During 90 days of storage at 4 °C and 25 °C, the samples maintained stable organoleptic properties, and no phase separation was observed. pH values ranged from 5.48 to 5.73 on the first day and from 5.61 to 5.89 after 90 days of storage.

The samples GISU70-3 (1.5% carbomer; 5% extract) and GISU70-4 (2.5% carbomer; 5% extract) were found to be the most promising, as they provide an optimal balance of rheological properties, structural stability, and uniform distribution of active ingredients.

Thus, the developed phytogel composition based on *I. scariosa* rhizome extract demonstrates promising physicochemical and technological characteristics and can be considered a basis for further pharmacological studies.

## Figures and Tables

**Table 1:** Bioactive compounds reported in *I. scariosa* and their distribution in other *Iris* species (literature data).

Compound	Molecular Formula	Class	Plant Part	Reported Biological Activity	Other *Iris* Species	Ref.
Apigenin	C_15_H_10_O_5_	Flavone	Rh, L	Antioxidant, anti-inflammatory	*I. schachtii*, *I. pseudacorus*	[26,27,28]
Artemisetin	C_20_H_20_O_8_	Flavone	Rh	-	-	[26]
Kanzakiflavone-2	C_16_H_10_O_6_	Flavone	Rh	Antioxidant	-	[26]
Hispidulin	C_16_H_12_O_6_	Flavone	Rh, L	Antioxidant, anti-inflammatory	*I. bungei*	[26,27,29]
Quercetin-3-glucoside	C_21_H_20_O_12_	Flavonol	Rh	Antioxidant, hepatoprotective	*I. pallida*, *I. germanica*	[26,27,30]
Rutin	C_27_H_30_O_16_	Flavonol	Rh	Antioxidant, anti-inflammatory	*I. schachtii*	[26,27,28]
Irisolone	C_17_H_12_O_6_	Flavonoid	Rh	Anti-inflammatory, cytotoxic	*I. adriatica*, *I. germanica*, *I. pallida*	[26,27]
Irilone	C_16_H_10_O_6_	Flavonoid	Rh	Cytotoxic, α-amylase inhibitor	*I. adriatica*, *I. germanica*	[26,27]
Iriflogenin	C_17_H_12_O_7_	Flavonoid	Rh	Cytotoxic	*I. dichotoma*	[26,27]
Pinocembrin	C_15_H_12_O_4_	Flavanone	Rh	Antioxidant, anti-inflammatory	-	[26]
Irigenin	C_18_H_16_O_8_	Isoflavone	Rh	Estrogen-like, anti-inflammatory	*I. adriatica*, *I. germanica*, *I. pallida*	[26,27]
Irisolidone	C_17_H_14_O_6_	Isoflavone	Rh	Antioxidant, anti-inflammatory	*I. germanica*	[26,27]
Genistein	C_15_H_10_O_5_	Isoflavone	Rh, R, L	Antioxidant, estrogen-like	*I. germanica*, *I. carthaliniae*, *I. lactea*	[26,27]
Tectorigenin	C_16_H_12_O_6_	Isoflavone	Rh	Antioxidant, antiproliferative	*I. adriatica*, *I. germanica*	[26,27]
Tectoridin	C_22_H_22_O_11_	Isoflavone glycoside	Rh	Anti-inflammatory	*I. hungarica*	[19,27]
Iriflophenone	C_13_H_10_O_5_	Xanthones	Rh, R, F	Antioxidant	*I. humilis*, *I. pumila*, *I. variegata*	[26,27]
4-O-methyliriflophenone	C_14_H_12_O_6_	Xanthones	Rh, R	Antibacterial	*I. pallida*, *I. Lactea*	[26,27]
Mangiferin	C_19_H_18_O_11_	Xanthones	Rh, F	Antioxidant, anti-inflammatory	*I. pallida*, *I. hungarica*, *I. sibirica*, *I. variegata*, *I. humilis*	[19,27,31]
Neomangiferin	C_19_H_18_O_11_	Xanthones	Rh	Antioxidant, antidiabetic	*I. adriatica*	[19,27,32]

Rh—rhizomes; R—roots; L—leaves; F—flowers.

**Table 2:** Chemical composition of *I. scariosa* essential oil determined by GC–MS.

No	Compound	Retention Time (min)	Area% ± SD	CV%	RI (est.)	RI (lit.)	Identification
1	Toluene	3.758	0.48 ± 0.01	2.7	760	769	MS
2	m-Xylene	5.210	0.54 ± 0.01	2.6	860	865	MS
3	α-Pinene	6.497	1.15 ± 0.08	6.5	930	932	MS
4	6-Methyl-5-hepten-2-one	6.960	3.08 ± 0.01	0.4	970	986	MS
5	β-Pinene	7.246	0.49 ± 0.02	4.6	980	979	MS
6	2-Ethyl-1-hexanol	7.873	0.39 ± 0.03	8.0	1020	1026	MS (tentative)
7	Di(cis-hex-3-enyl) fumarate	8.291	1.10 ± 0.01	0.6	1050	n.d.	MS (tentative)
8	2-Nonanone	8.973	3.67 ± 0.01	0.3	1100	1096	MS
9	Camphor	9.160	13.11 ± 0.75	5.7	1140	1141	MS
10	Decalin derivative	9.842	0.37 ± 0.01	1.8	1160	n.d.	MS (tentative)
11	Octanoic acid	10.414	7.27 ± 0.01	0.1	1175	1170	MS
12	Terpinen-4-ol	10.788	1.08 ± 0.01	1.0	1185	1174	MS
13	Naphthalene	10.854	0.73 ± 0.01	1.0	1190	1180	MS
14	α-Terpineol/related alcohol methanol	10.964	2.06 ± 0.01	0.4	1195	1189	MS (tentative)
15	Bicyclic monoterpene alcohol	11.096	0.89 ± 0.03	2.8	1198	n.d.	MS (tentative)
16	Dodecane	11.349	1.65 ± 0.01	0.3	1200	1200	MS
17	2,6-Dimethylundecane	11.635	0.88 ± 0.01	0.7	1225	1220	MS (tentative)
18	trans-bicyclodecane derivative	11.965	0.40 ± 0.01	1.4	1245	n.d.	MS (tentative)
19	Heptylcyclohexane	12.108	0.96 ± 0.01	0.7	1255	1240–1260	MS
20	4-Methyldodecane	12.417	0.74 ± 0.06	8.6	1265	1265	MS (tentative)
21	2-Methyldodecane	12.494	0.63 ± 0.01	1.5	1270	1268	MS
22	3-Methyldodecane	12.615	0.51 ± 0.01	2.3	1273	1273	MS
23	2-Undecanone	12.670	2.55 ± 0.01	0.3	1275	1293	MS
24	2-Methoxy-4-vinylphenol	12.923	1.32 ± 0.01	0.6	1290	1310	MS
25	Tridecane	13.110	9.79 ± 0.01	0.1	1300	1300	MS
26	1,1′-Bicyclohexyl	13.341	0.63 ± 0.01	0.9	1320	1310–1330	MS (tentative)
27	Eugenol	13.693	0.80 ± 0.01	0.3	1335	1356	MS
28	n-Decanoic acid	13.880	16.11 ± 0.01	0.1	1345	1364	MS
29	Tetradecane	14.782	0.40 ± 0.01	3.3	1400	1400	MS
30	Sesquiterpene hydrocarbon	15.860	0.34 ± 0.01	2.5	1450	1450–1500	MS (tentative)
31	Esterified monoterpene	16.817	0.78 ± 0.01	0.4	1480	1470–1500	MS (tentative)
32	Dodecanoic acid (Lauric acid)	17.004	16.38 ± 0.01	0.1	1500	1470–1500	MS
33	Ethyl dodecanoate	17.543	2.45 ± 0.01	0.3	1550	1540–1560	MS

Identification: MS—identified by mass spectral matching (NIST library); tentative—tentative identification based on spectral similarity. RI (est.)—approximate retention indices estimated based on the elution of n-alkanes (C12–C14) detected in the chromatogram. Due to the limited number of reference alkanes, these values should be considered as indicative only. RI (lit.)—literature retention indices for non-polar columns (HP-5MS type), based on [33]. n.d.—not determined.

**Table 3:** Analytical parameters of HPLC method for phenolic compounds.

No.	Compound	Linear Range (mg/L)	R^2^	LOD (mg/L)	LOQ (mg/L)
1.	Gallic acid	6.125–67.375	0.9993	1.07	3.25
2.	Catechin	6.125–67.375	0.9995	0.61	1.84
3.	Epicatechin	6.125–67.375	0.9994	0.65	1.97
4.	Naringin	6.125–67.375	0.9995	0.50	1.51
5.	Phlorizin	6.125–67.375	0.9994	0.68	2.07

**Table 4:** Semi-quantitative (estimated) HPLC determination of phenolic compounds in dense extract of *I. scariosa*, rhizomes (*n* = 3).

No. Peak	Compound	tR (min) M ± SD	Estimated Content (mg/g) M ± SD	RSD (%)	Contribution (%)
1	Gallic acid	4.197 ± 0.005	48.077 ± 0.14	0.29	44.2
2	Catechin	8.331 ± 0.002	7.954 ± 0.05	0.63	7.3
3	Epicatechin	10.346 ± 0.003	49.118 ± 0.07	0.14	45.2
6	Rutin	12.736 ± 0.005	0.530 ± 0.00	0.02	0.49
4	Naringin	17.732 ± 0.003	1.495 ± 0.01	0.67	1.37
5	Phlorizin	19.066 ± 0.006	1.502 ± 0.002	0.13	1.38

**Table 5:** Composition of model gel formulations (% *w*/*w*).

Components	Function	Samples				
		GISU70-1	GISU70-2	GISU70-3	GISU70-4	GISU70-5
*I. scariosa* rhizome extract	Active ingredient	2.5	3.5	5.0	5.0	5.0
*I. scariosa* essential oil	Active ingredient	3.0	3.0	3.0	3.0	3.0
Carbopol 940	Gelling agent	1.0	1.0	1.5	2.5	3.2
Glycerin	Humectant	28.0	28.0	28.0	28.0	28.0
NaOH solution (10%)	Neutralizer	q.s.	q.s.	q.s.	q.s.	q.s.
Polysorbate 80 (Tween 80)	Dispersing agent	0.3	0.3	0.3	0.3	0.3
Purified water	Solvent	q.s. to 100	q.s. to 100	q.s. to 100	q.s. to 100	q.s. to 100

All compositions are expressed as % *w*/*w*. Volumetric components were converted to mass units based on their densities to ensure accuracy and reproducibility.

**Table 6:** Evaluation of organoleptic properties of model gel formulations under different storage conditions.

Parameters	GISU70-1	GISU70-2	GISU70-3	GISU70-4	GISU70-5
Color	Light brown	Light brown	Brownish	Brownish	Brownish
Odor	Characteristic odor of the extract	Characteristic odor of the extract	Characteristic odor of the extract	Characteristic odor of the extract	Characteristic odor of the extract
Appearance	Fluid, transparent formulation with good spreadability	Gel-like, plastic, transparent formulation with good spreadability	Gel-like, homogeneous, transparent formulation with good spreadability	Gel-like, homogeneous, transparent formulation with good spreadability	Jelly-like, homogeneous, transparent formulation with good spreadability
Homogeneity	Homogeneous, no phase separation observed	Homogeneous, no phase separation observed	Homogeneous, no phase separation observed	Homogeneous, no phase separation observed	Homogeneous; no phase separation observed
Storage at 25 ± 2 °C (1 day)	No visible changes	No visible changes	No visible changes	No visible changes	No visible changes
Storage at 25 ± 2 °C (90 days)	No visible changes	No visible changes	No visible changes	No visible changes	No visible changes
Storage at 4 ± 2 °C (1 day)	No visible changes	No visible changes	No visible changes	No visible changes	No visible changes
Storage at 4 ± 2 °C (90 days)	No visible changes	No visible changes	No visible changes	No visible changes	No visible changes
Centrifugation/cyclic stability test (6 cycles)	Slight decrease in viscosity	No visible changes	No visible changes	No visible changes	Increase in structural density

**Table 7:** Evaluation of pH values of model gel formulations over a 90-day storage period.

Samples		GISU70-1	GISU70-2	GISU70-3	GISU70-4	ISU70-5
pH	Day 1	5.61 ± 0.04	5.73 ± 0.06	5.48 ± 0.03	5.67 ± 0.04	5.73 ± 0.06
	Day 90	5.70 ± 0.06	5.77 ± 0.02	5.61 ± 0.03	5.77 ± 0.05	5.89 ± 0.05

**Table 8:** Chemiluminescence parameters in ROS (I) and LPO (II) models under the action of the tested samples.

Substance	Model	Light Sum, a.u.	Spontaneous Luminescence, a.u.
Control	I	29.4 (27.5–30.1)	1.4 (1.3–1.5)
	II	24.7 (23.4–27.8)	1.1 (1.0–1.1)
Ethanol extract of *Iris scariosa* rhizomes (IS-U 70%)	I	14.3 (12.7–16.1) *	0.9 (0.8–1.0) *
	II	20.1 (17.8–21.3) *	0,9 (0.7–1.0) *
GISU70–3 (gel)	I	13.1 (10.5–14.3) *	0.8 (0.7–1.1) *
	II	23.2 (20.7–25.8)	1.3 (1.0–1.3)
70% Ethanol	I	10.3 (8.9–12.5) *	1.2 (1.2–1.3)
	II	11.4 (10.6–13.8) *	1.3 (1.1–1.3) *
Sunflower oil	I	16.5 (13.8–17.6) *	1.6 (1.5–1.7) *
	II	15.4 (13.7–17.2) *	1.1 (0.9–1.2)
Ascorbic acid	I	4.5 (4.3–4.8) *	0.9 (0.9–1.1) *
	II	5.6 (5.4–5.8) *	1.2 (1.2–1.3) *

Data are presented as median (Q1–Q3) or mean (minimum–maximum), as indicated in each table; *n* = 6. Asterisks (*) indicate statistically significant differences compared to the control (*p* ≤ 0.05). Comparisons versus reference substances, such as ascorbic acid, are indicated where applicable (*p* < 0.05). All luminescence values are expressed in arbitrary units (a.u.) where relevant.

**Table 9:** Effect of the developed gels on paw edema diameter in mice after formalin-induced inflammation (mm).

Group	0 h	4 h	24 h
Control	3.2 (2.9–3.4)	4.5 (4.3–4.7) ^a,†^	3.7 (3.5–4.0) ^a,β,†^
GISU-4	2.9 (2.7–3.2)	3.8 (3.7–4.0) *^,a^	3.2 (2.9–3.3) *^,β^
GISU-3	3.4 (2.9–3.5)	3.7 (3.6–4.0) *^,a^	3.2 (3.0–3.3) *^,β^
Diclofenac sodium	3.0 (2.7–3.2)	3.5 (3.3–3.9) *^,a^	3.2 (2.9–3.4) *

Data are presented as median (interquartile range). *n* = 6 animals per group. * *p* < 0.05 vs. control group at the corresponding time point. ^a^ *p* < 0.05 vs. baseline (0 h) within the same group. ^β^ *p* < 0.05 vs. 4 h within the same group. ^†^ *p* < 0.05 vs. diclofenac sodium group.

## Data Availability

The original contributions presented in this study are included in the article. Further inquiries can be directed to the corresponding authors.
